# Applicability of the methylated CpG sites of paired box 5 (PAX5) promoter for prediction the prognosis of gastric cancer

**DOI:** 10.18632/oncotarget.1973

**Published:** 2014-05-14

**Authors:** Jingyu Deng, Han Liang, Rupeng Zhang, Qiuping Dong, Yachao Hou, Jun Yu, Daiming Fan, Xishan Hao

**Affiliations:** ^1^ Department of Gastroenterology, Tianjin Medical University Cancer Hospital, City Key Laboratory of Tianjin Cancer Center and National Clinical Research Center for Cancer, Tianjin, China; ^2^ Central laboratory, Tianjin Medical University Cancer Hospital, City Key Laboratory of Tianjin Cancer Center and National Clinical Research Center for Cancer, Tianjin, China; ^3^ Institute of Digestive Disease, Li Ka Shing Institute of Health Science, Chinese University of HongKong, Shatin, HongKong; ^4^ State Key Laboratory of Cancer Biology and Institute of Digestive Diseases, Xijing Hospital, Fourth Military Medical University, Xi'an, China

**Keywords:** Paired box 5, Methylation, Prognosis, Gastric cancer

## Abstract

Paired box gene 5 (PAX5), a member of the paired box gene family, is involved in control of organ development and tissue differentiation. In previous study, PAX5 promoter methylation was found in gastric cancer (GC) cells and tissues. At present study, we found that the inconsistently methylated levels of PAX5 promoter were identified in the different GC tissues. The methylated CpG site count and the methylated statuses of four CpG sites (-236, -183, -162, and -152) were significantly associated with the survival of 460 GC patients, respectively. Ultimately, the methylated CpG -236 was the optimal prognostic predictor of patients identified by using the Cox regression with AIC value calculation. These findings indicated that the methylated CpG -236 of PAX5 promoter has the potential applicability for clinical evaluation the prognosis of GC.

## INTRODUTION

In addition to Japan and Korea, China is the third highest incidence of gastric cancer (GC). With 42% of worldwide cases occurring, GC has been a major health burden in China [1]. Due to lack of the highly specific biomarkers of carcinogenesis and prognostic evaluation, the overall survival (OS) of GC is still dismal regardless of the improvements of therapeutic techniques [2]. PAX5, a member of the paired box gene family, is involved in control of organ development and tissue differentiation [3]. Although researchers reported that PAX5 expression was detected in non-hematopoietic tumors such as neuroendocrine tumors, Merkel cell carcinoma, and mesonephric tumors [3, 4], Kolhe et al [5] demonstrated that pulmonary small cell carcinoma was negative PAX5 expression of the immunohistochemical staining. Several researchers demonstrated that the methylation of PAX5 DNA promoter was frequently found in human malignancies [6-8]. In previous study, our collaborators demonstrated that PAX5 is a novel functional tumor suppressor in GC and PAX5 promoter methylation was relative to the survival of GC [8]. In view of the small scale patients and only the qualitatively detective method of the promoter methylation in that study, we intended to detect the quantitatively methylated levels of PAX5 DNA promoter in the large scale patients for elaborate elucidation the prognostic predicted value of PAX5 promoter methylation in GC.

## RESULTS

### Patient Demongraphics

All 460 GC patient clinicopathological characteristics are listed in Table 1. The median OS of all patients was 21 months. Of 460 patients, 61 (13.26%) were alive when the follow-up was over.

**Table 1:** Patient information

Gender		
Male	315 (68.48%)	
Female	145 (31.52%)	
Age at surgery		
≤ 60	272 (59.13%)	
> 60	188 (40.87%)	
Tumor size		
< 4.0	66 (14.35%)	
≥ 4.0	394 (85.65%)	
Tumor location		
Upper third	113 (24.57%)	
Middle third	118 (25.65%)	
Lower third	201 (43.70%)	
More than 2/3 stomach	28 (6.08%)	
Depth of tumor invasion (T stage)		
T1	5 (1.09%)	
T2	46 (10.00%)	
T3	285 (61.96%)	
T4	124 (26.95%)	
Number of metastatic lymph nodes (N stage)		
N0	111 (24.13%)	
N1	163 (35.44%)	
N2	107 (23.26%)	
N3	79 (17.17%)	
Location of lymph node metastasis		
No	111 (24.13%)	
Perigastric	158 (34.35%)	
Extragastric	191 (41.52%)	
Lauren classification		
Intestinal	122 (26.52%)	
Diffuse	320 (69.57%)	
Mixed	18 (3.91%)	
Methylated CpG site count		
1 or less	193 (41.96%)	
2 or more	267 (58.04%)	
Methylated status of CpG -236		
Unmethylated	356 (77.39%)	
Methylated	104 (22.61%)	
Methylated status of CpG -183		
Unmethylated	301 (65.43%)	
Methylated	159 (34.57%)	
Methylated status of CpG -162		
Unmethylated	349 (75.87%)	
Methylated	111 (24.13%)	
Methylated status of CpG -152		
Unmethylated	284 (61.74%)	
Methylated	176 (38.26%)	

### Protein and mRNA Expression of PAX5 in GC Tissues and Normal Gastric Mucosal Tissues

**Figure 1:**
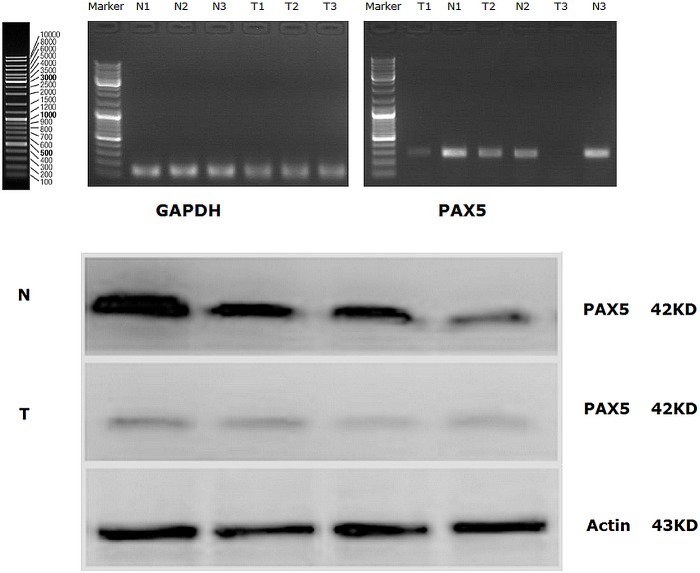
(A) PAX5 mRNA expression (RT-PCR) in GC tissues and in normal gastric mucosal tissues; (B) Western Blot analysis for PAX5 protein expression in GC tissues and in normal gastric mucosal tissues. (Representation: T, GC tissues; N, normal gastric mucosal tissues).

PAX5 mRNA expression was detected in 25 of 460 GC tissues and 25 normal gastric mucosal tissues by Semi-quantitative reverse transcription polymerase chain reaction (RT-PCR) (Figure 1A). We also found there were significant differences of PAX5 mRNA expression in 25 GC tissues. The mean value of relative mRNA expression of PAX5 in 25 GC tissues was 0.863±0.357, while the mean value of relative mRNA expression of PAX5 in 25 normal gastric mucosal tissues was 1.759±0.821. The mean value of relative mRNA expression of PAX5 in 25GC tissues was lower than that in 25 normal gastric mucosal tissues (P =0.036).

Similarly, PAX5 protein expression was also detected in 25 of 460 GC tissues and 25 normal gastric mucosal tissues by Western blot, simultaneously (Figure 1B). We found there were significant differences of PAX5 protein expression in 25 GC tissues. The mean value of relative protein expression of PAX5 in 25 GC tissues was 0.517±0.204, while the mean value of relative protein expression of PAX5 in 25 normal gastric mucosal tissues was 1.704±0.658. The mean value of relative protein expression of PAX5 in 25 GC tissues was much lower than that in 25 normal gastric mucosal tissues (P =0.009).

### Methylation Detection of PAX5 Promoter

**Figure 2:**
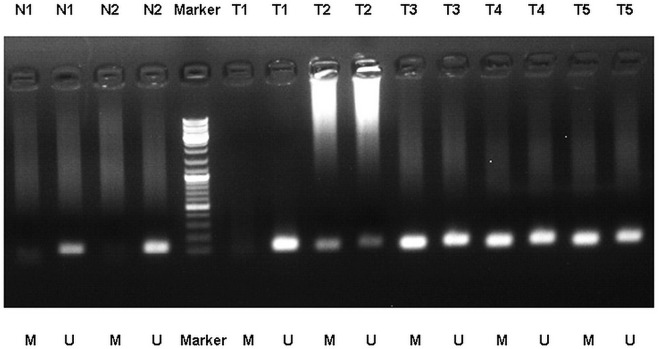
MSP detection of PAX5 promoter methylation in different GC tissues and normal gastric mucosal tissues. (Representation: T, GC tissues; N, normal gastric mucosal tissues; M, methylated; U, unmethylated).

We detected the different levels of PAX5 promoter methylation (including methylation, non-methylation, and partial methylation) in 25 of 460 GC tissues with the methylation-specific PCR (MSP) analysis, while no PAX5 promoter methylation was found in 25 normal gastric mucosal tissues (Figure 2). Of 25 GC tissues, 6 (24%) presented with the methylation of PAX5 promoter, 16 (64%) presented with the partial methylation of PAX5 promoter, and 3 (12%) presented with the non-methylation of PAX5 promoter.

**Figure 3:**
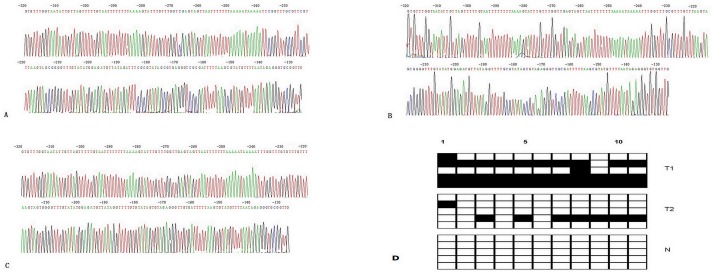
(A) Bisulphite sequencing figure of PAX5 in GC tissue 1, (B) Bisulphite sequencing figure of PAX5 in GC tissue 2, (C) Bisulphite sequencing figure of PAX5 in normal gastric mucosal tissue, and (D) Bisulfite sequencing results in GC tissues and in normal gastric mucosal tissue. (Representation: T, GC tissues; N, normal gastric mucosal tissues).

Subsequently, we adopted the bisulphite genomic sequencing (BGS) analyzed the methylated statuses of all CpG sites of PAX5 promoter to obtain the precisely quantitatively methylated levels of PAX5 promoter in all 460 GC patients. Methylated CpG site count of 460 GC patients ranges between 0 and 11. Of the 460 patients included in the study, 361 patients (78.48%) presented with one or more methylated CpG sites and 99 patients (21.52%) presented with no methylated CpG site. Although GC patients without methylated CpG site had slightly median OS than those with one or more methylated CpG sites (21 vs 20 months), there is not statistical difference between two groups of patients (P =0.995). According to the result of cut-point analysis for the methylated CpG site count, 267 patients (58.04%) presented with two or more methylated CpG sites and 193 patients (41.96%) presented with one or zero methylated CpG sites. No methylated CpG site was found in the normal gastric mucosal epithelial tissues. The methylation sequencing pictures and CpG site charts were shown in Figure 3.

### Survival Analysis

**Figure 4:**
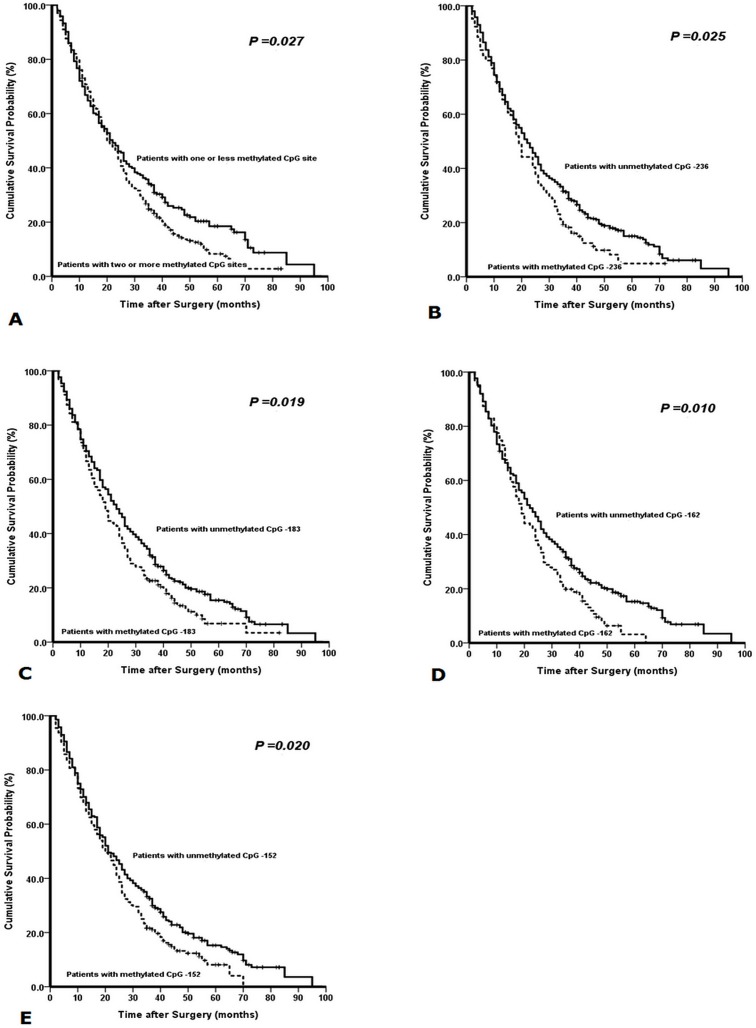
Kaplan-Meier survival curves comparing months of survival in gastric cancer patients are shown for (A) methylated CpG site count of PAX5 promoter, (B) methylated status of CpG -236, (C) methylated status of CpG -183, (D) methylated status of CpG -162, and (E) methylated status of CpG -152.

With the univariate survival analysis, four clinicopathological characteristics were found to have statistically significant associations with OS of 460 GC patients. They were as follows: T stage (P < 0.001), N stage (P < 0.001), tumor size (P =0.030), and tumor location (P =0.050) (Table 2). We also demonstrated that the methylated statuses of four CpG sites (CpG -236, CpG -183, CpG -162, and CpG -152) of PAX5 promoter had significant association with the survival of 460 GC patients (P =0.025, =0.019, =0.010, and =0.020), respectively. In addition, we found that the methylated CpG site count (P =0.027) was significantly associated with the OS of patients with the Kaplain-Meier curves discrimination (Table 2) (Figure 4).

**Table 2:** Survival analysis of 460 GC patients

Variables	Median OS (mo)	X2 value	Univariate P value	HR value	Multivariate P value	AIC value
Gender						
Male	22	0.573	0.449			
Female	20					
Age at surgery (years)						
≤ 60	20	0.107	0.744			
> 60	23					
Tumor location						
Upper third	21	7.829	0.050			
Middle third	18					
Lower third	24					
≥ 2/3 stomach	16					
Tumor size (cm)						
< 4.0	26	4.694	0.030			
≥ 4.0	20					
Lauren classification						
Intestinal	26	5.708	0.058			
Diffuse	20					
Mixed	17					
Depth of tumor invasion (T stage)						
T1	70	34.445	< 0.001	1.476 (1.245-1.750)	< 0.001	76.964
T2	27					
T3	24					
T4	12					
Number of metastatic lymph nodes (N stage)						
N0	37	98.121	< 0.001	1.575 (1.422-1.744)	< 0.001	94.722
N1	23					
N2	18					
N3	10					
Location of lymph node metastasis						
No	37	48.676	< 0.001			
Perigastric	19					
Extragastric	17					
Methylated CpG site count						
1 or less	22	4.915	0.027			
2 or more	20					
Methylated status of CpG -236						
Unmethylated	22	5.015	0.025	1.393 (1.103-1.761)	0.005	74.384
Methylated	19					
Methylated status of CpG -183						
Unmethylated	23	5.517	0.019			
Methylated	19					
Methylated status of CpG -162						
Unmethylated	22	6.702	0.010			
Methylated	19					
Methylated status of CpG -152						
Unmethylated	21	5.398	0.020			
Methylated	20					

All above eight factors were included in a multivariate Cox proportional hazards model with bootstrapping performance to adjust for the effects of covariates. With the multivariate analysis, the independent predictors with the OS of all 460 GC patients were identified to be the methylated status of CpG -236 (HR =1.393, P = 0.005), N stage (HR =1.552, P < 0.001) and T stage (HR =1.559, P <0.001) (Table 2).

Lastly, we demonstrated that the methylated status of CpG -236 of PAX5 promoter had the smaller AIC value than anyone of other independent predictors calculated within the Cox proportional hazard regression model, representing the optimal prognostic predictor of GC (Table 3).

**Table 3:** Correlation analysis between methylated status of CpG sites of PAX5 promoter and clinicopathological characteristics

Variables	Methylated CpG sites ≥ 2	Methylated CpG -236	Methylated CpG -183	Methylated CpG -162	Methylated CpG -152
Male	182	71	111	78	120
Female	85	33	48	33	56
P value	0.865	0.958	0.655	0.641	0.914
≤ 60	154	53	93	66	106
> 60	133	51	66	45	70
P value	0.456	0.054	0.839	0.935	0.706
Upper third	66	34	43	31	49
Middle third	79	32	50	37	52
Lower third	109	34	61	39	70
≥ 2/3 stomach	13	4	5	4	5
P value	0.086	0.020	0.032	0.047	0.031
< 4.0	36	16	18	12	20
≥ 4.0	231	88	141	99	156
P value	0.534	0.732	0.178	0.222	0.151
Intestinal	75	29	43	33	49
Diffuse	180	70	111	73	122
Mixed	12	5	5	5	5
P value	0.458	0.792	0.821	0.606	0.598
T1	3	1	1	0	2
T2	25	10	15	8	13
T3	170	62	97	64	109
T4	69	31	46	39	52
P value	0.835	0.905	0.817	0.079	0.447
N0	63	27	39	25	38
N1	95	34	58	44	67
N2	58	26	34	21	36
N3	51	17	28	21	35
P value	0.551	0.873	0.922	0.506	0.320
No	63	27	39	25	38
Perigastric	91	34	49	36	60
Extragastric	113	17	71	50	78
P value	0.911	0.863	0.479	0.687	0.521

## DISCUSSION

PAX proteins function as nuclear transcription factors important for cellular differentiation, migration, and proliferation [9]. These molecules have been considered as the strong transcriptional regulators and targets for disruption in oncogenesis [9]. Pax5, an important B cell lineage commitment factor in the early stages of B cell development, is essential for B cell lineage commitment [10]. In addition to the hematopoietic tumors, PAX5 expression was detected in several solid malignacies [3-5]. Torlakovic et al [11] reported that Pax-5 was expressed regularly in poorly differentiated neuroendocrine tumors, benign and malignant mesonephric tissues, and müllerian duct-derived tumors. Authors considered that those results were important for correct interpretation of results in immunophenotyping of undifferentiated tumors, for diagnosis of mesonephric carcinoma, and, potentially, for correct classification of neuroendocrine tumors in small biopsy samples [11]. Dong et al [4] reported the expression of PAX5 in neuroendocrine carcinomas such as Merkel cell carcinoma (93.5%) and small cell carcinoma (73.3%). Other researchers demonstrated that the low or loss expression of PAX5 was detected in the human cancers, which indicates PAX5 may function as a potential tumor suppressor in the carcinogenesis [5-8]. Methylation of promoter CpG islands is one type of epigenetic modification that regulates gene expression, and the hypermethylation of promoter CpG islands of tumor suppressor genes has been identified in many different cancers [12]. Recently, authors demonstrated that PAX5 DNA promoter methylation, resulting in the silencing of PAX5, was frequently detected in the several malignancies and was negatively associated with the poor clinical outcome of patients [6-8, 13, 14]. Palmisano et al [13] demonstrated that inactivation of the PAX5 β gene likely contributed to neoplastic development by inhibiting growth regulation through effects on CD19 gene expression. They also proposed that methylation of the PAX5genes was found only in tumors and surrounding tissue, and only rarely in normal epithelial cells, which supported their evaluation as intermediate markers in lung and breast cancer for improvement the sensitivity and specificity to develop risk models for detecting these cancers [13]. Similarly, we also found that the silencing of PAX5 in the GC tissues and the methylation of PAX5 promoter was specific in the GC tissues. Therefore, we thought that PAX5 functioned as a tumor suppressor gene in GC.

In 2011, our collaborators reported that PAX5, a functional tumor suppressor involved in liver carcinogenesis through direct regulation of the p53 signaling pathway, was frequently inactivated by promoter methylation in primary hepatocellular carcinoma tissues [7]. Last year, Li et al [8] reported that the downregulation of PAX5 was closely linked to the promoter hypermethylation status and the ectopic expression of PAX5 in silenced GC cell lines (AGS and BGC823) inhibited colony formation and cell viability, arrested cell cycle, induced apoptosis, suppressed cell migration and invasion and repressed tumorigenicity in nude mice. They also demonstrated that PAX5 hypermethylation was detected in 77% (144 of 187) of primary GC tissues, which was significantly associated with the poor survival of GC patients [8]. At present study, we also found that mRNA and protein expression of PAX5 was down-regulated in the GC tissues, and the inconsistent levels of PAX5 promoter methylation were exclusively detected in GC tissues by using the MSP method. However, we think that it is important to confirm the levels of PAX5 promoter methylation for further elucidation the values of PAX5 DNA methylation in canceration and prognostic evaluation of GC.

In this study, the occurrence rate of PAX5 promoter methylation (78.48%) in the 460 GC patients was similar to the finding of previous study [8]. Many researchers proposed that the methylated CpG site detection was appropriate for precisely quantitative evaluation the correlation between the methylated levels of gene promoter and the various abnormally biological events [15-18]. Subsequently, we meticulously analyzed the methylated CpG sites of PAX5 promoter in 460 GC patients by using the BGS method with no less than five positive clones for each GC sample. We found that four methylated CpG sites (CpG -236, CpG -183, CpG -162, and CpG -152) of PAX5 promoter were significantly associated with the poor survival of 460 GC patients, respectively. Besides, we also demonstrated that patients with two or more methylated CpG sites of PAX5 promoter had significantly shorter survival than those with one or zero methylated CpG sites of PAX5 promoter.

In addition to N stage and T stage, the methylated status of CpG -236 was also identified to be the independent predictor of survival of 460 GC patients by mean of the Cox regression with forward step procedures. Owing to its smaller AIC value, the methylated status of CpG -236 was ultimately demonstrated to be the optimal predictor of GC patients' prognosis by using the AIC value calculation within the Cox regression. Finally, we found that only primary tumor location was correlated to the methylated statuses of CpG sites of PAX5 promoter (Table 3). That is to say, patients with primary tumor located in lower third stomach should detect the methylated statuses of CpG sites of PAX5 promoter, especially in the CpG sites (-236, -183, -162, and -152), for contribution to enhancement the precise efficiency of the prognostic evaluation.

## PATIENTS AND METHODS

### Data Source

After approval from the Tianjin Medical University Cancer Hospital institutional review board, data from the cancer registry of the Tianjin Cancer Institute was obtained. Oral and written inform consents were obtained from the patients who were included in this study. Information which was obtained through participating cancer registry included: age, gender, tumor location, tumor size, depth of tumor invasion (T stage, according to the sixth edition UICC TNM classification for GC), number of metastatic lymph nodes (N stage, according to the sixth edition UICC TNM classification for GC), extent of lymph node metastasis, Lauren classification, and follow-up vital status.

### Patients and Study Samples

For PAX5 promoter methylation analysis, we collected 460 fresh GC tissues from GC patients who underwent curative gastrectomy between April 2003 and December 2007 at the Department of Gastroenterology, Tianjin Medical University Cancer Hospital. In addition, a cohort of 25 normal gastric mucosal epithelial tissues derived from normal people between 2004 and 2007 at the Department of Endoscopic Examination and Treatment, Tianjin Medical University Cancer Hospital. All the tumor and normal gastric mucosal epithelial tissues were histologically verified, and were used to detect the methylated status of PAX5 promoter by means of the bisulphite sequencing (BSP). Of 460 GC tissues, 25 were used to detect PAX5 expression and detect qualitative methylation of PAX5 DNA by means of the methylation-specific PCR (MSP).

All patients were not administered radiation, chemical or biological treatment prior to the potentially curative gastrectomy. Adjuvant chemotherapy or radiotherapy was not routinely administrated in patients routinely. The clinicopathological characteristics of these 460 GC patients are summarized in Table 1. The patients' consent was obtained for the use of the tissue samples and records, and the study protocol was approved and permission for use of the clinical data was given by the Institutional Research Ethics Committee of Tianjin Medical University Cancer Hospital.

### Surgical Treatment

Curative resection was deﬁned as a complete lack of grossly visible tumor tissue and metastatic lymph nodes remaining after resection, with pathologically negative resection margins. Primary tumors were resected en bloc with limited or extended lymphadenectomy (D1 or D2-3 according to the Japanese Gastric Cancer Association (JGCA)). Surgical specimens were evaluated as recommended by the sixth UICC TNM classification for GC.

### DNA extraction and RNA extraction

Genomic DNA was extracted from 460 GC tissues and 25 normal gastric mucosal tissues using QIAamp DNA mini kit (Qiagen, Valencia, CA) following the manufacturer's instructions. Sodium bisulphite modification of genomic DNA was performed by using the EZ DNA Methylation-GoldTM Kit (Zymo Research, Hornby, Canada). RNA was extracted from 25 of 460 GC tissues and 25 normal gastric mucosal tissues using Trizol reagent (Invitrogen, Carlsbad, CA) according to the manufacturer's instructions.

### Western Blotting Analysis

25 of 460 GC tissues and 25 normal gastric mucosal tissues were added to 1 mL of 100 mmol/L Tris/HCl (pH 7.5), 100 mmol/L NaCl, 0.5% sodium deoxycholate, 1 mmol/L ethylenediaminetetraacetic acid, 1% Nonidet P-40, 0.1% sodium dodecyl sulfate, and protease inhibitor, respectively. After blocking, 50 ug sample was incubated for 60 minutes with a rabbit anti- PAX5 (Abcam, ab92512, 1:1000 dilution) at room temperature. Gel Imager system (Asia Xingtai Mechanical and Electrical Equipment Company, Beijing, China) to analyze images and to determine gray values.

### Semi-quantitative Reverse Transcription Polymerase Chain Reaction (RT-PCR) Analysis

The expression of PAX5 mRNA was detected by RT–PCR in 25 of 460 GC tissues and 25 normal gastric mucosal tissues. Total RNA was reversely transcribed to cDNA in a 20 ul volume by using Reverse Transcription kit (Invitrogen, Carlsbad, CA). Primers designed and utilized for PAX5 was as follows: Forward sequence: 5′-CCTGAAGGTCCGACTGAGAAG-3′, and Reverse sequence: 5′-GATACTAAAGAAGGAGGGATGAGC-3′. The GAPDH gene was used as an endogenous control for semi-quantitative DNA-PCR. Primers designed and utilized for GAPDH was as follows: Forward sequence: 5′-GAAGGTGAAGGTCGGAGTC-3′, and Reverse sequence: 5′-GAAGATG GTGATGGGATTTC-3′. The PCR Cycling conditions for all sequences were 35 cycles of denaturation at 95°C for 3 minutes, annealing at 94°C for 30 seconds, and extension at 56°C for 30 seconds followed by a final extension at 72°C for 8 minutes. All PCR product electrophoreses were performed on a 2% agarose gel with ethidium bromide and visualized using the Gel Imager system (Asia Xingtai Mechanical and Electrical Equipment Company, Beijing, China).

### Sodium Bisulfite Treatment

Sodium bisulphite modification of genomic DNA was performed by using the EZ DNA Methylation-GoldTM Kit (Zymo Research, Hornby, Canada).

### Methylation-Specific PCR (MSP)

25 of 460 GC tissues and 25 normal gastric mucosal tissues were detected the qualitatively methylated analysis of PAX5 promoter with the methylation-specific PCR (MSP). PAX5 primers detecting methylated (M) or unmethylated (U) alleles of the PAX5 promoter were: PAX5-MF, 5′-AAATAAAAATTCGGTTTGCGTTC-3′ and PAX5-MR, 5′-AAACATACGC TTAAAAATCGCG-3′ for methylated alleles; PAX5-UF, 5′-TAAAAATAAAA ATTTGGTTTGTGTTTG-3′ and PAX5-UR, 5′-CCTCTATTAAAACATACACT TAAAAATCACA-3′ for unmethylated alleles. MSP was performed for 25 cycles using Ampli Taq-Gold (methylation-specific primer, annealing temperature 600°C; unmethylation specific primer, annealing temperature 580°C). MSP primers were firstly checked for not amplifyling any unbisulfited DNA and the specificity of MSP was further confirmed by direct sequencing of some PCR products. PCR reactions were resolved on a 2% agarose gel.

### Bisulphite Genomic Sequencing (BGS)

All 460 GC tissues and 25 normal gastric mucosal tissues were detected the qualitatively methylated analysis of PAX5 promoter with the bisulphite genomic sequencing (BGS). Hot start PCR with the bisulfite-treated DNA was performed with a 197bp PCR product spanning promoter region −321bp to -130bp relative to the transcription start site of PAX5. 11 CpG sites were contained in the promoter region of PAX5. The sequences of PCR primers were as follows: F: 5′-GTGTTTGGTAATATTGTTAGTTTTTGTAAT-3′; R: 5′-CAACCRCACCCTCTATTAAAACATAC-3′. The purified PCR products were cloned into the pUC18-T vector (Biodee, Beijing, China), and no less than five positive clones for each sample were randomly selected and sequenced by Shanghai Sangon Co. (Shanghai, China).

### Follow-Up

After curative surgery, all patients were followed every 3 or 6 months for 2 year at outpatient department, every year from the third to fifth years, and then annually thereafter until the patient died. The median follow-up for the entire cohort was 44 months (range: 2-104). The follow-up of all patients who were included in this study was completed in December 2012. Ultrasonography, CT scans, chest X-ray, and endoscopy were obtained with every visit.

### Statistical Analysis

The median OS was determined by using the Kaplan-Meier method, and log-rank test was used to determine significance. Factors that were deemed of potential importance on univariate analyses (P <0.05) were included in the multivariate analyses. Multivariate analysis of OS was performed by means of the Cox proportional hazards model with forward step procedures. Hazard ratios (HR) and 95% CI were generated. The Akaike information criterion (AIC) value within a Cox proportional hazard regression model was calculated for different variables to measure theirs prognostic prediction ability. A smaller AIC value indicates a better model for predicting outcome [19]. With the cut-point survival analysis [20], the optimal cutoff for CpG site conut was identified to be one. Significance was defined as P < 0.05. All statistical analyses were performed with SPSS 18.0 software.
